# Physiological responses of the co-cultivation of PGPR with two wheat cultivars *in *vitro under stress conditions

**DOI:** 10.1186/1753-6561-8-S4-P108

**Published:** 2014-10-01

**Authors:** Camila Gazolla Volpiano, Andressa Estevam, Kléber Saatkamp, Fernando Furlan, Eliane Cristina Gruszka Vendruscolo, Marise Fonseca Dos Santos

**Affiliations:** 1 Universidade Federal do Paraná, Palotina, Brazil; 2 UNIOESTE - Departamento de Agronomia, Foz do Iguaçu, Brazil

## 

Wheat (*Triticum aestivum *L.) may be exposed to different stress conditions that influence its productivity. In southern Brazil the rainfed wheat crop is limited by dry spells consisting of short periods of drought during rainy periods. One way to confer tolerance to the drought´s effects and stimulate plant productivity is the action of a group of bacteria capable of making association with plants, known as Plant Growth Promoter Bacteria (PGPB), as *Herbaspirillum seropedicae *and *Azospirillum brasilense*, both diazotrophic. It was demostrated that the PGPB can benefit the plants in several ways: synthesizing some phytohormones, siderophores, biological nitrogen fixation, inducing systemic resistance etc. The strain *H. seropedicae *SmR1 unlike *A. brasilense *AbV5, presents a gene encoding the 1-aminocyclopropane-1-carboxylic acid (ACC) deaminase, which breaks ACC, the ethylene precursor in alpha-keto-butyric acid (AKB) and ammonium ion. The indole-3-acetic acid (IAA) is a phytohormone, produced by PGPB that modulates the synthesis of plant ethylene, and it is known as stress and senescence promoter. The IAA can stimulate the synthesis of ACC by increasing the activity of ACC synthase and inhibit the transformation of ACC into ethylene by decreasing the activity of ACC oxidase. The aim of this work was study the *in vitro *interactions of two varieties of wheat, CD120 and an ancestor cultivar "Frontana", with the *H. seropedicae *SmR1 and *A. brasilense *AbV5 to verify the physiological changes in the culture medium under osmotic stress imposed by polyethylene glycol (PEG 6000). Wheat plantlets were obtained throughout mature embryos cultured on solid MS medium, aseptically transferred after 28 days to 10 mL of 1/10 MS liquid medium with or without PEG and inoculated with *H. seropedicae *and/or *A. brasilense*. The controls consisted of wheat plantlets with and without PEG in the same condition. After 5 days, plantlets and bacteria were harvested and the co-culture medium was taken to determine the concentrations of IAA (Glickmann & Dessaux, 1995) [1], AKB (Penrose and Glick, 2003) [2], and total protein (Hartree, 1972) [3]. The treatments of CD120 without PEG and with *H. seropedicae *showed the highest IAA concentration. In Frontana the amounts of IAA are lower as compared to CD120, however, treatments under the stress condition (with PEG) and *A. brasilense *stood out. Treatments with PEG and both bacteria showed similar performances associated with CD120. The amounts of AKB increased in the presence of PEG and *H. seropedicae *for both cultivars, probably by action of ACC deaminase activity. The total protein increased in the presence of PEG comparing the two controls for both cultivars. The total protein in CD120 was greater only when bacteria were present compared to their controls indicating a plant cell death for CD120 and Frontana. It is possible to conclude that the association of *H. seropedicae *is more effective with CD120 and indicated the reduction of ethylene. Frontana associates better with *A. brasilense *Possibly PEG causes cell death in the system or exudation of proteins by the cultivars tested. These data corroborate with the literature where plant vs. bacteria interaction is genotype and strain dependent.
